# Extracellular Vesicles: Biological Packages That Modulate Tumor Cell Invasion

**DOI:** 10.3390/cancers15235617

**Published:** 2023-11-28

**Authors:** Madison Schmidtmann, Crislyn D’Souza-Schorey

**Affiliations:** Department of Biological Sciences, University of Notre Dame, Notre Dame, IN 46556, USA

**Keywords:** cancer, extracellular vesicles, extracellular matrix, exosomes, microvesicles, tumor cell invasion, metastasis

## Abstract

**Simple Summary:**

Cell invasion is an intrinsic cellular behavior wherein cells respond to various signals and bring about the degradation of the extracellular matrix (ECM) to facilitate their movement through surrounding tissues. In cancer, this ‘invasive’ behavior is aberrantly increased when cells from the primary tumor invade local tissues and blood vessels leading to metastasis. The past couple of decades have witnessed much progress in the understanding of the molecular and cellular mechanisms that underlie invasion of the ECM by tumor cells. A growing body of evidence implicates extracellular vesicles (EVs)—small membrane-enclosed sacs shed by tumor cells—as modulators of tumor cell invasion. These sacs carry important molecules that affect ECM degradation and composition. In this article, we review the various ways by which EVs can modify the ECM to bring about cell invasion and metastases.

**Abstract:**

Tumor progression, from early-stage invasion to the formation of distal metastases, relies on the capacity of tumor cells to modify the extracellular matrix (ECM) and communicate with the surrounding stroma. Extracellular vesicles (EVs) provide an important means to regulate cell invasion due to the selective inclusion of cargoes such as proteases and matrix proteins into EVs that can degrade or modify the ECM. EVs have also been shown to facilitate intercellular communication in the tumor microenvironment through paracrine signaling, which can impact ECM invasion by cancer cells. Here, we describe the current knowledge of EVs as facilitators of tumor invasion by virtue of their effects on proteolytic degradation and modification of the ECM, their ability to educate the stromal cells in the tumor microenvironment, and their role as mediators of long-range communication aiding in cell invasion and matrix remodeling at secondary sites.

## 1. Introduction

Cell invasion, which is the movement of cells through the surrounding extracellular matrix (ECM), is an intricately regulated process that is critical to many normal physiological processes including during embryonic development, wound repair, and immune surveillance [1]. The acquisition of invasive potential is also a necessary step during cancer initiation and metastasis [2]. Metastasis is a complex process that includes invasion of primary tumor cells through the ECM and stroma, intravasation into the bloodstream, extravasation, and distant growth of tumor cells in various organs other than the primary site [3]. There is now compelling evidence that the organization and composition of the three-dimensional stromal ECM markedly influences tumor cell invasion and involves both cell autonomous and non-cell autonomous regulation [4]. 

Tumor cells adopt several modes of invasion in response to cues from the extracellular environment including the ECM. Single-cell invasion has been observed in tumor cells adopting amoeboid or mesenchymal-like morphologies [5]. Tumor cells can toggle between these morphological phenotypes in response to the stiffness and complexity of the ECM [6,7,8]. Amoeboid cells assume a rounded shape and exhibit high membrane blebbing. Cells with a mesenchymal phenotype are spindle-shaped or flat and fibroblast-like and develop invadopodia (proteolytic actin protrusions formed at the ventral surface of the cell) and other cell protrusions [6,9]. Tumor cells with both phenotypes, amoeboid and mesenchymal, are capable of matrix metalloproteinase (MMP)-mediated extracellular matrix degradation and invasion [10]. Confinement by the extracellular matrix and matrix compliance appears to determine invasion capacity. In this regard, protease-mediated invasion increases when the pore size of the ECM decreases below the limit of deformation for the cell nucleus that is sufficient for invasion through ECM by mechanical force [11,12]. In addition to single-cell movement, tumor cells also use multicellular streaming and collective cell invasion, which is reminiscent of cell migration during organ morphogenesis. Collective cell movement is distinct from single-cell motility, wherein the movement pattern of multiple cells retain cell–cell connections and migrate coordinately [13]. During collective cell invasion, cells can adopt leader and follower cell signatures unique to their roles to effectively facilitate this mode of invasion [9,14]. Leader cells respond to cues from the ECM, soluble factors, and chemoattractants generating navigable tracks for follower cells through intracellular communication and the release of proteases such as cathepsin B [14].

**Figure 1:**
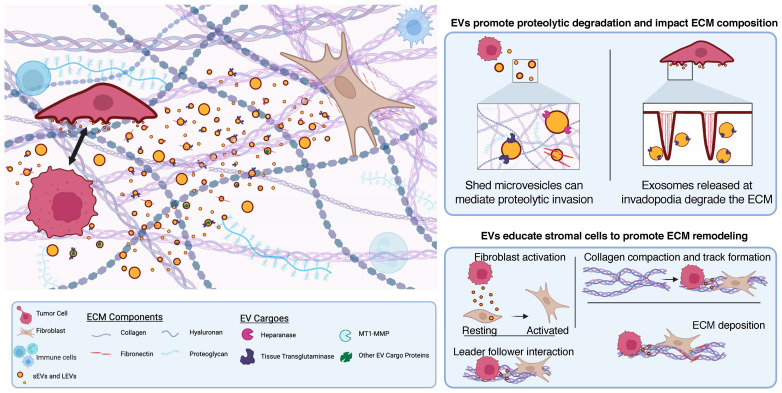
Extracellular vesicles (EVs) shed from cells facilitate invasion of the extracellular matrix (ECM) in many ways. Tumor cells may toggle between amoeboid and mesenchymal phenotypes and shed EVs that contain proteases such as MMPs and heparinase, as well as ECM components like fibronectin, allowing for matrix remodeling and cell invasion. Highly aggressive tumor cell lines adopt amoeboid morphologies and release larger-sized ectosomes to facilitate degradation. Exosomes are secreted at invadopodia on the adherent surface of tumor cells to aid in proteolytic degradation of the ECM. EVs can deposit ECM components that affect matrix composition and stiffening. Shed EVs may also activate stromal cells such as fibroblasts and inform behaviors that advance tumorigenesis. In response to EV uptake, fibroblasts increase collagen compaction and provide tracks for leader–follower pathfinding behaviors during matrix invasion. EV uptake also results in an increase in fibronectin production by fibroblasts. Created with BioRender.com.

Extracellular vesicles (EVs) broadly encompass a heterogenous population of membrane-enclosed sacs released from nearly all cell types. Once regarded as cell debris, we know now that EVs are shed by cells from all organisms via multiple mechanisms of biogenesis that appear to be evolutionarily conserved. Depending on the cell of origin, EVs may contain numerous cellular cargoes including various RNA species, DNA, lipids, metabolites, signaling molecules, and cell-surface receptors [15,16,17,18]. EVs may be classified as either small EVs or large EVs on the basis of size [19]. Small EVs include exosomes, <80 nm vesicles that are formed as intraluminal vesicles in multivesicular bodies (MVBs) and released into the extracellular space upon fusion of the limiting membrane of the MVB with the plasma membrane (PM), and arrestin domain-containing protein 1-mediated microvesicles that pinch from the cell surface. The small EVs are less than 200 nm in diameter. Microvesicles, large oncosomes, migrasomes, and apoptotic bodies, all of which bud from the plasma membrane and are greater than 200 nm in size, are categorized as large EVs or ectosomes. Tumor-derived EVs have been hypothesized to promote cell-to-cell communication due to their ability to “educate” other cells locally or in distant tissues/organs. Cargoes packaged in EVs secreted by a ‘donor’ cell may be released into the extracellular space or transferred to another cell leading to phenotypic changes in the ‘recipient’ cell [15,16,17,18]. In the case of the latter, EVs bind to surface molecules on recipient cells and trigger changes at the cell surface of the recipient or upon internalization. EVs may serve to facilitate cargo exchange, deposit paracrine information, present decoys, or rid the cell of unwanted material. These collective roles of EVs have been shown to have marked effects on epithelial/endothelial barrier function, fibroblast reprogramming, and immune cell activation during tumor progression [20,21,22]. This review centers on EVs as important modulators of extracellular matrix invasion by tumor cells. As we describe below, EVs facilitate direct alteration of the ECM through proteolytic degradation, deposition of ECM components, or by educating stromal cells to modify the ECM (Figure 1), all of which facilitate tumor cell invasion. 

## 2. Extracellular Vesicles Contribute to Extracellular Matrix Alterations Impacting Tumor Cell Invasion

### 2.1. EV Cargo Results in Proteolytic Degradation of the ECM

Extracellular vesicles are known to contain proteases that are involved in proteolytic degradation of the extracellular matrix. Membrane type 1-matrix metalloproteinase (MT1-MMP) has been found as bioactive cargo in several classes of EVs [23,24]. Substrate targets of MT1-MMP include aggrecan, collagen types I, II, and III, fibronectin, gelatin, laminin-1 and -5, and vitronectin [24,25]. In addition to ECM substrates, MT1-MMP also cleaves pro-MMP2 and pro-MMP13 to remove the pro-peptide, resulting in activation of these enzymes [24,26]. 

MT1-MMP is released into the extracellular space by shed EVs in both ameboid and mesenchymal modes of cell invasion. During ameboid cell invasion, MT1-MMP is delivered to nascent microvesicles forming at the cell surface, a process regulated by vesicle-associated membrane protein 3 (VAMP3) association with the tetraspanin, CD9 [10]. In contrast, vesicle-associated membrane protein 7 (VAMP7), which is required for the delivery of MT1-MMP to invadopodia and functional degradation of the matrix [27], is not present on microvesicles [27]. Knockdown of VAMP3, but not VAMP7, affected the delivery of MT1-MMP to microvesicles. In contrast, cellular depletion of VAMP7 but not VAMP 3 affected protease delivery to invadopodia. Proteases in shed microvesicles allow for cell invasion through compliant matrices and cross-linked collagen matrices [6,10]. Functional MT1-MMP has also been found in exosomes. Isolated exosomes contain both the pro form and active MT1-MMP, and these exosomes were able to activate MMP-2 and degrade collagen I and gelatin [28]. Interestingly, exosome secretion has been linked to invadopodia biogenesis in a synergistic relationship where exosome cargoes aid in invadopodia formation, stabilization, and proteolytic activity [29].

The formation of microvesicles as well as exosomes has been linked to signaling downstream of ADP ribosylation factor 6 (ARF6) [30,31], and thus common regulators may be involved in the formation of various EV subtypes. ARF6 is the small GTP-binding protein that has been shown to regulate endosomal trafficking and actin cytoskeletal remodeling, and as such its GTP/GDP cycle regulates cellular processes as varied as epithelial cell adhesion [32], invadopodia formation [33,34] and cytokinesis [35,36], all of which are accompanied by changes in cell shape and morphology. Interestingly in this regard, EVs have been implicated in all these processes [29,37,38,39]. This supports the contention that EV biogenesis pathways may be triggered by and depend on cellular context.

In addition to exosomes and microvesicles, large oncosomes (approximately 1–10 μm) have also been shown to contain proteolytically active matrix metalloproteases [40,41,42]. Finally, migrasomes are larger-sized EVs formed at the tips or intersections of retraction fibers at the back of migrating cells [43,44]. While a role for migrasomes in proteolytic degradation is not described, migrasome release is reported to be highly correlated with the migration of cells.

**Figure 2:**
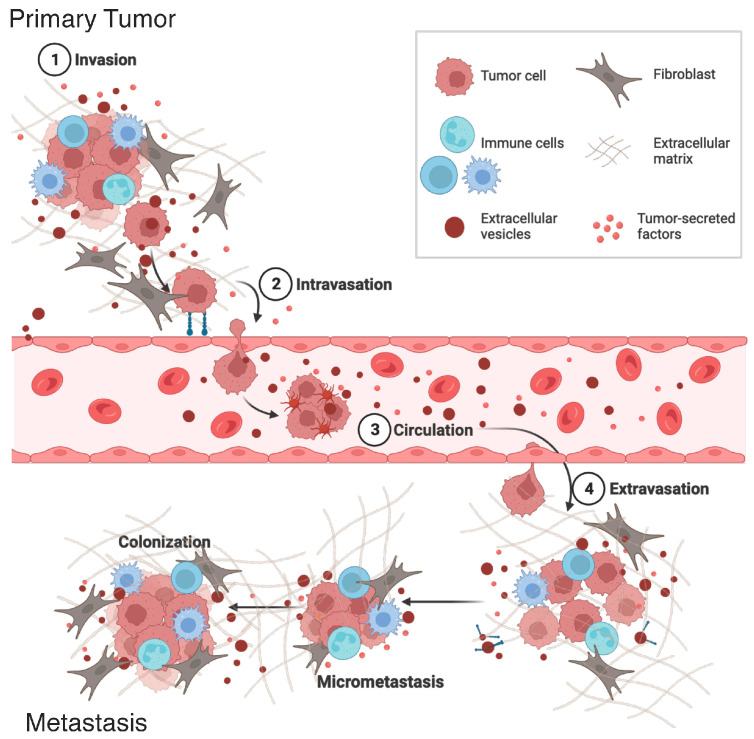
EVs influence tumor invasion at various stages of cancer progression. Tumor-derived EVs at the primary tumor site facilitate early tumor invasion. EVs carry cargoes to direct organ-specific metastasis and can also impact recipient cell behavior and ECM remodeling at secondary sites enabling colonization and metastasis. Created with BioRender.com.

In addition to facilitating local invasion, tumor-derived EVs also facilitate ECM remodeling to facilitate movement toward and colonization at distant sites (Figure 2). EVs are found in the blood stream and lymphatic system, allowing for long-range communication [45,46,47]. This builds upon Stephen Paget’s proposed “seed and soil” theory, where cancer cells metastasize to specific organs and condition metastatic sites before colonization to form a pre-metastatic niche (PMN). In preparation for successful metastasis, the PMN acquires several key features including the activation of resident stromal cells, affecting the immune landscape, and remodeling of the ECM [46]. A study by Deep et al. found that intraperitoneal treatment of hypoxia-derived EVs promotes the formation of a PMN. Here, there was increased matrix metalloproteinase activity at metastatic sites allowing for ECM remodeling, along with levels of fibronectin and collagen in a prostate cancer model [48]. Thus, EVs have a proteolytic role in local invasion as well as invasion of tumor cells at distal sites.

### 2.2. EVs Contribute to ECM Composition to Promote Invasion

The ECM plays a dynamic role in the creation of the tumor microenvironment (TME), and its dysregulated remodeling can promote tumorigenesis. Tumor-promoting alterations to the ECM such as a deposition of fibrillar collagen or increased deposition of fibronectin are associated with worse patient prognosis [42,49,50]. While tumor cells and tumor-associated stromal cells affect ECM composition through matrix deposition, tumor-derived EVs also contain ECM components that can be deposited in the tumor microenvironment. For example, fibronectin is present in exosomes secreted by breast, glioma, and fibrosarcoma tumor cells [51,52,53]. Fibronectin-positive exosomes were shown to be required for directional and persistent movement of fibrosarcoma cells, wherein exosome secretion promoted fibronectin-dependent adhesion [52]. Fibronectin is present on the outside of the exosomes, providing the correct topology to promote adhesion and invasion. In another study, fibronectin and laminin found in microvesicles during trophoblast migration are important for implantation efficiency [54]. Here, laminin and fibronectin interact with integrins to activate c-Jun N-terminal Kinase (JNK) and focal adhesion kinase (FAK), stimulating trophoblast migration and increasing implantation efficiency [54]. These studies highlight the importance of EVs for ECM alteration in normal, healthy processes, and suggest cancer cells hijack basic EV processes during disease progression.

The ECM and stroma surrounding solid tumors are often stiff as a result of higher amounts of ECM proteins, increased remodeling, and cross-linked ECM proteins [55,56]. Changes to the tumor microenvironment through modification of the ECM can drastically affect the progression of disease and propensity for invasive cell behaviors. Tumor-derived EVs can alter tissue microstructure resulting in stiffening and changes in genes associated with ECM and fibronectin protein expression [53]. During breast tumorigenesis, collagen crosslinking results in stiffened ECM that induces invasion [57]. Furthermore, breast tumor cells secrete an increased number of exosomes in response to ECM stiffness [58]. These exosomes, dubbed stiffness-tuned exosomes, are released in response to stiffened ECM and are required for cell contractility necessary for cell spreading and motility in stiff matrices. Additionally, stiffness-tuned exosomes increased cell attachment and contain thrombospondin-1, which not only plays a role in cell–cell adhesion but also in cell-ECM adhesion [57]. Together these studies point to an important role for EVs in promoting and responding to tumor stiffness that advances tumorigenesis.

In addition to structural proteins, the extracellular matrix contains heparan sulfate proteoglycans embedded within the matrix. Heparan sulfate is a side chain found on proteoglycans and is degraded by heparinase [59,60]. Exosomes secreted in response to chemotherapy treatment contain a high level of heparanase on the surface of the exosome. Exosomal heparanase is transferred to other cells, increasing their expression of active heparanase and stimulating ERK signaling [61]. Notably, increased abundance of heparanase is associated with chemoresistance, and targeting heparanase improves patient outcomes [62].

## 3. Tumor-Derived Extracellular Vesicles Educate Stromal Cells to Form Pro-Invasive Microenvironments

As previously discussed, tumor EVs contain ECM components that affect the composition of the matrix. EVs produced by tumor cells are also able to affect the ECM by delivering cargoes to other cell types. The tumor microenvironment is host to resident and infiltrating cells. Cells such as macrophages, fibroblasts, and vascular endothelial cells play significant roles in remodeling the extracellular matrix and TME to promote tumor cell invasion, advancing tumorigenesis [63]. Due to the variety of cargo found in EVs, tumor cells are able to strategically alter recipient cells to promote tumorigenesis—effects range from metabolic shifts to altered immune response and evasion [16,64,65]. The section below focuses largely on the tumor cell–fibroblast interactions shown to promote cell invasion and ECM remodeling.

Fibroblasts are particularly susceptible to behavioral alterations as a result of EV uptake. Fibroblasts are somewhat ambiguously defined as interstitial cells of mesenchymal lineage and are not of epithelial, endothelial, or immune cell origin [66]. Cancer-associated fibroblasts (CAFs) are fibroblasts located adjacent to or within a tumor and populations are predominately composed of local resident fibroblasts rather than non-local precursors [66,67]. Normal fibroblasts participate in wound healing, response to tissue damage, and alter the ECM deposition and structure [66,68]. CAFs coopted for ECM alterations contribute to collagen and fibronectin deposition, and restructure the ECM to allow tumor cell invasion.

Extracellular vesicle cargo can change ECM composition and stiffness through stromal recipient cell behavior to favor invasive phenotypes. For instance, as a result of microvesicles shed from tumor cells, recipient fibroblasts are transformed to alter the ECM. Exosomes containing mutant p53 have been reported to influence ECM architecture and tumor cell invasion by promoting integrin recycling in fibroblasts that result in a less adhesive, more branched ECM network [69].

EVs have also been shown to carry the protein crosslinking enzyme tissue transglutaminase and the ECM component fibronectin [51]. These components are transferred to fibroblasts, resulting in activation of the fibroblasts to promote a pro-tumorigenic microenvironment. Alternatively, fibroblasts found at the periphery of tumors, where the matrix is most stiff, are activated in response to EVs shed by aggressive breast cancer cells. This causes fibroblasts to increase cell spreading, traction forces, and collagen compaction [70]. Further, while highly migratory breast cells are capable of single-cell migration, it has been shown that weakly migratory cells require microvesicle-mediated communication with fibroblasts for invasion. Weakly migratory cells release microvesicles that activate murine fibroblasts and promote cancer cell movement in a tumor spheroid model [71]. In a colorectal cancer model, fibroblasts activated by tumor EVs invade the ECM, creating a track for the tumor cells to follow. In this case, the fibroblasts act as leader cells in response to the secreted vesicles by the colorectal tumor cells and allow the cells to invade the local tissue without undergoing epithelial-to-mesenchymal transition (EMT) [72]. The enrichment of EVs in adhesion molecules serves to bind surrounding ECM [23], whereas soluble chemoattractants in EVs [73] may serve as guidance cues facilitating leader–follower movement. These are all examples of how tumor EVs educate stromal cells such as fibroblasts to alter the ECM facilitating pro-invasive phenotypes and worsening disease outcomes. Furthermore, as stated above, other cell types found in the tumor microenvironment, such as macrophages, also take up EVs with influence on the TME [74].

## 4. Conditioning of the Pre-Metastatic Niche ECM by Extracellular Vesicles

The formation of the pre-metastatic niche (PMN) is a result of conditioning organ tissue environments to support metastatic growth. The ECM within the PMN is altered in response to several factors including soluble proteins secreted by the primary tumor, coopted stromal cells, and cells recruited to the niche such as macrophages and fibroblasts [75,76]. Extracellular vesicles play an important role in facilitating these processes due to their ability to intravasate and extravasate the vascular and lymphatic systems allowing for long-range communication, potentially providing a means of metastatic organotropism (Figure 2). For instance, exosomes derived from breast cancer cell sub-lines containing integrins α_6_β_5_ and α_6_β_1_ were associated with lung metastasis, while exosomes displaying α_v_β_5_ were associated with liver metastasis. Interestingly, the EV integrin profiles did not necessarily match the tumor cell integrin profiles [77]. Another study found that breast and lung tumor-derived exosomes contain cell migration-inducing and hyaluronan-binding protein (CEMIP) and that CEMIP+ exosomes promote brain organotropism by affecting angiogenesis and encouraging an inflammatory microenvironment [78]. This helps to explain why specific cancers typically metastasize to certain organs and potentially provides a means to identify the risk of metastasis through EV biopsy. Additionally, EVs may modulate the PMN by influencing resident cells. One study found that exosomes are preferentially taken up by resident cells of metastatic destinations in vitro and in vivo and that these EVs colocalize with specific cell types in different ECM environments [79]. The study also found that circulating exosomes display integrin expression profiles that could be used to predict metastasis sites.

Extracellular vesicles can also impact the ECM composition of the pre-metastatic niche by modulating recipient cell behavior. Stromal cells in the TME can accomplish this through the deposition of matrix components [49]. The uptake of PDAC-derived exosomes by Kupffer cells results in transforming growth factor β (TGF-β) secretion and an increase in fibronectin production by hepatic stellate cells. The PDAC-derived exosomes contained macrophage migration inhibitory factor, and this correlated with patients who later developed liver metastasis [79]. In the case of metastatic melanoma, tail vein injection of EVs secreted by the SW1 melanoma cell line resulted in the deposition of fibronectin within the lungs accompanied by an increase in CD45+ cells [80]. Ghoshal et al. found that this was a result of insulin-like growth factor 2 mRNA binding protein 1 (IGF2BP1) and this is likely due to the IGF2BP1 effect on cargo transport by these EVs [80].

Additional ECM components, such as tenascin-C, influence the formation of the pre-metastatic niche. Tenascin-C is a large ECM glycoprotein, typically downregulated in healthy adult tissues [81]. However, overexpression is frequently observed in cancer at the invasive tumor front, implicating tenascin-C in tumor progression and metastasis [81,82,83]. In a bladder cancer model, tumor-derived EVs induced tenascin-C expression by fibroblasts, and this is associated with a worse prognosis. The induction of tenascin-C expression at the pre-metastatic niche is potentially a result of EV-contained cytokines [84]. Furthermore, tumor-derived EVs are able to pass through the blood–brain barrier via transcytosis, and these EVs interact with astrocytes to condition the PMN by downregulating expression of the tissue inhibitors of MMPs-2 (TIMP2) [85,86]. This decrease in TIMP expression corresponded with increased ECM modulation and astrocyte migration [85]. Altogether, these studies point toward the need to further understand EV influence on stromal cells to better understand the metastatic process.

## 5. Concluding Remarks

Tumor-derived EVs are recognized largely as mediators of cell-to-cell communication through the horizontal transfer of bioactive cargoes—protein, nucleic acids, and lipids—between cells. Significantly, cargoes in EVs can also influence the extracellular matrix with an impact on disease progression. As described above, EVs can facilitate amoeboid or mesenchymal modes of invasion, extracellular matrix (ECM) remodeling, directional movement, and the education of stromal cells to impact tumor invasion at various points during disease progression. In addition to local invasion at the primary tumor, EVs affect the pre-metastatic niche to promote a pro-metastatic microenvironment and provide a long-range means for primary tumor cells to advance disease through invasion and metastasis. An important future direction will be to identify EV cargoes and cellular regulators involved in EV-mediated ECM alteration and communication within the pre-metastatic niche. A better understanding of the role of both, the cellular context and extracellular cues, in generating various EV types during matrix invasion is also needed. In addition, how the functional roles of EVs in tumor invasion may impact disease progression is not well understood and remains an important but understudied area of cancer biology. Further studies along these lines represent promising avenues for a more complete understanding of the mechanisms involved in tumor invasion, the conditioning of the pre-metastatic niche during metastasis, and for designing new strategies for therapeutic intervention.
